# Supplementary material for: Deficiency in Inactive Rhomboid Protein2 (iRhom2) Alleviates Alcoholic Liver Fibrosis by Suppressing Inflammation and Oxidative Stress
Source: Int J Mol Sci. 2022 Jul 12;23(14):7701. doi: 10.3390/ijms23147701 (PMC9317380; doi:10.3390/ijms23147701)
Supplement: Supplementary file 1 [file ijms-23-07701-s001.zip › ijms-1745240-supplementary.pdf]

Supplementary table

Table S1. Cell viability of L02 and HSC-t6 induced by different concentrations of alcohol for 4 h

Cell	Alcoholic medium concentration(%)	A-value	Cell viability(%)
L02	0	0.561±0.021	100
	0.5	0.544±0.015	96.98
	1.0	0.524±0.031	93.35
	1.5	0.502±0.039	89.42
	2.0	0.492±0.034	87.70
	2.5	0.446±0.015	79.48
	3.0	0.389±0.016	69.29
HSC-t6	0	0.426±0.059	100
	0.5	0.407±0.094	95.69
	1.0	0.392±0.027	92.05
	1.5	0.374±0.015	88.03
	2.0	0.369±0.055	86.77
	2.5	0.322±0.075	75.68
	3.0	0.291±0.035	68.48

Table S2. sgRNA sequences

gene	Sense（5’-3’）
1 iRhom2 F	CACCGGTTTGGAGTCAGCGGCGACT
1 iRhom2 R	AAACAGTCGCCGCTGACTCCAAACC
2 iRhom2 F	CACCGCGCCGCAAGAGAATGTCTGT
2 iRhom2 R	AAACACAGACATTCTCTTGCGGCGC
3 iRhom2 F	CACCGACGTATGGCATCGCACCCGT
3 iRhom2 R	AAACACGGGTGCGATGCCATACGTC

Table S3. L02 gene sequence

L02	Sequence（5'-3'）
GAPDH F	GAGAAGGCTGGGGCTCATTT
GAPDH R	AGTGATGGCATGGACTGTGG
iRhom2 F	ATTTGCACGTATGGCATCGC
iRhom2 R	TCTCCAGGTCCCTCAGGATG
TACE F	GGGCAGAGGGGAAGAGAGTA
TACE R	CTCCTGGCACTTCTTCTGGG
IL-6 F	TTCGGTCCAGTTGCCTTCTC
IL-6 R	CAGCTCTGGCTTGTTCCTCA
IL-18 F	TGGCTGCTGAACCAGTAGAAG
IL-18 R	GAGGCCGATTTCCTTGGTCA
TACE F	GGGCAGAGGGGAAGAGAGTA
TACE R	CTCCTGGCACTTCTTCTGGG
IL-1β F	TGAGCTCGCCAGTGAAATGA
IL-1β R	AGATTCGTAGCTGGATGCCG
TNF-α F	GTGACAAGCCTGTAGCCCAT
TNF-α R	CAGACTCGGCAAAGTCGAGA
TNFR1 F	CTGGAGCTGTTGGTGGGAAT
TNFR1 R	GTTCCTTCAAGCTCCCCCTC
TNFR2 F	CCAGGTGGCATTTACACCCT
TNFR2 R	CAGGAAGGAGGTGCTTGGAG
HO-1 F	GTGCCACCAAGTTCAAGCAG
HO-1 R	CAGCTCCTGCAACTCCTCAA
Nrf2 F	CTCCACAGAAGACCCCAACC
Nrf2 R	TCTGCAATTCTGAGCAGCCA
SOD F	ACAAAGATGGTGTGGCCGAT
SOD R	AACGACTTCCAGCGTTTCCT

CAT F	ACCAAGGTTTGGCCTCACAA
CAT R	AGATCCGGACTGCACAAAGG

Table S4. HSC-t6 gene sequence

HSC-t6	Sequence（5’-3’）
GAPDH F	GCGAGATCCCGCTAACATCA
GAPDH R	CTCGTGGTTCACACCCATCA
iRhom2 F	TGGAGGAGGATGCTGTCGAT
iRhom2 R	AACGAACGTCAGCCAGTAGG
TACE F	ACCACTTTGGTGCCTTTCGT
TACE R	GTTCAGCTCGCCTCTTCACT

Table S5. Mouse gene sequence

Mouse	Sequence（5’-3’）
GAPDH F	CATCACTGCCACCCAGAAGACTG
GAPDH R	ATGCCAGTGAGCTTCCCGTTCAG
IL-6 F	TTTCCTCTGGTCTTCTGGAGT
IL-6 R	TCTGTGACTCCAGCTTATCTCTTG
IL-1β F	TGGACCTTCCAGGATGAGGACA
IL-1β R	GTTCATCTCGGAGCCTGTAGTG
IL-18 F	ACTTTGGCCGACTTCACTGT
IL-18 R	GTCTGGTCTGGGGTTCACTG
TNF-α F	GGTGCCTATGTCTCAGCCTCTT
TNF-α R	GCCATAGAACTGATGATAGGGAG
iRhom2 F	TGGAGGAGGATGTGGTCGAT
iRhom2 R	TGTGGGATCCCTCGGAAGTA

Supplementary graph

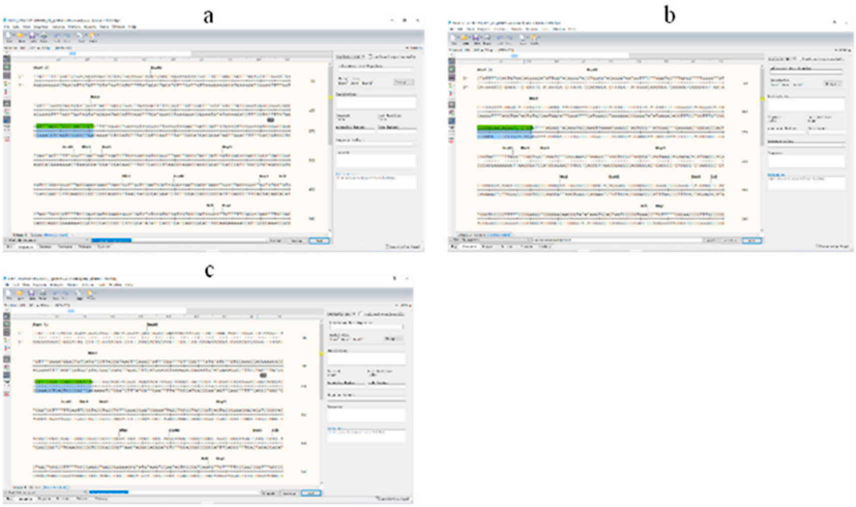

Figure S1. Comparison with sgRNA sequences. (a), (b) and (c) Three different sequence plasmids were successfully constructed.

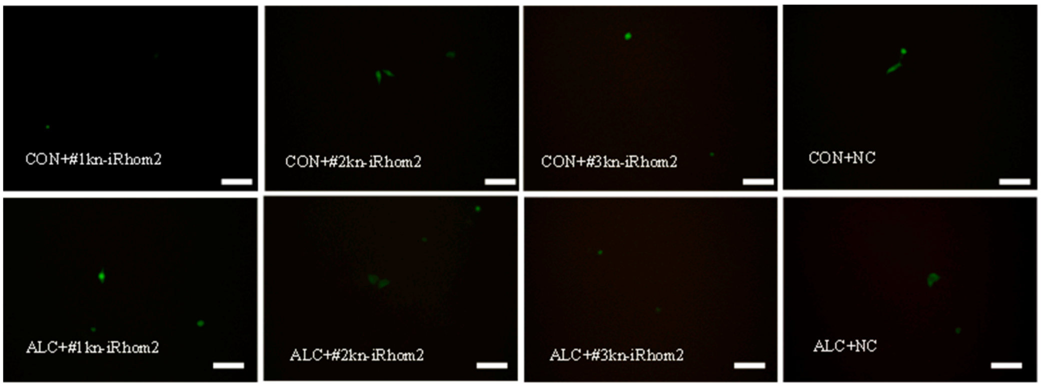

Figure S2. Cell transfection
